# Risk factors for atrial fibrillation in dogs with dilated cardiomyopathy

**DOI:** 10.3389/fvets.2023.1183689

**Published:** 2023-05-09

**Authors:** Carlo Guglielmini, Carlotta Valente, Giovanni Romito, Chiara Mazzoldi, Marco Baron Toaldo, Marlos Goncalves Sousa, Marcela Wolf, Tamyris Beluque, Oriol Domenech, Valentina Patata, Francesco Porciello, Paolo Ferrari, Domenico Caivano, Barbara Contiero, Helen Poser

**Affiliations:** ^1^Department of Animal Medicine, Production and Health, University of Padua, Legnaro, Italy; ^2^Department of Veterinary Medical Sciences, Alma Mater Studiorum - University of Bologna, Bologna, Italy; ^3^Department of Veterinary Medicine, Federal University of Paraná, Curitiba, Brazil; ^4^Department of Cardiology, AniCura Istituto Veterinario Novara, Novara, Italy; ^5^Department of Veterinary Medicine, University of Perugia, Perugia, Italy

**Keywords:** arrhythmia, canine, cardiac disease, electrocardiography, echocardiography, heart failure

## Abstract

**Introduction:**

Atrial fibrillation secondary to dilated cardiomyopathy (DCM) frequently affects large-breed dogs. The aim of the present study was to identify risk factors for the development of atrial fibrillation in dogs of different breeds with an echocardiographic diagnosis of DCM.

**Methods:**

In this multicenter retrospective study, we searched the electronic databases of five cardiology referral centers for dogs with an echocardiographic diagnosis of DCM. A comparison of clinical and echocardiographic variables was performed between dogs developing atrial fibrillation and those not developing atrial fibrillation and the ability to distinguish between these two groups of dogs was evaluated by receiver operating characteristic curve analysis. Univariate and multivariable logistic regression analysis estimated the odds ratio (OR) with 95% confidence interval (CI) of developing atrial fibrillation.

**Results:**

We included 89 client-owned dogs with occult and overt echocardiographic DCM. Of these, 39 dogs (43.8%) had atrial fibrillation, 29 dogs (32.6%) maintained a sinus rhythm, and 21 dogs (23.6%) showed other cardiac arrhythmias. Left atrial diameter had high accuracy (area under the curve = 0.816, 95% CI = 0.719–0.890) to predict the development of atrial fibrillation at the cut-off of >4.66 cm. After multivariable stepwise logistic regression analysis, only increased left atrial diameter (OR = 3.58, 95% CI = 1.87–6.87; *p* < 0.001) and presence of right atrial enlargement (OR = 4.02, 95% CI = 1.35–11.97; *p* = 0.013) were significant predictors of atrial fibrillation development.

**Discussion:**

Atrial fibrillation is a common complication of DCM in the dog and is significantly associated with increased absolute left atrial diameter and right atrial enlargement.

## 1. Introduction

Atrial fibrillation (AF) is the most common pathological supraventricular arrhythmia in humans and different animal species, including dogs and horses (1–4). According to the underlying cardiac condition, AF can be classified in primary or lone, when no recognizable cardiac disease is present and secondary AF, when a structural heart disease is associated with the arrhythmia (2, 5). This latter is certainly the most common type of AF in the dog, although some large breed dogs (e.g., Irish wolfhound) can present AF without recognizable structural cardiac disease (3, 5, 6).

Left atrial enlargement and remodeling are probably the major risk factors for AF development in both humans and animals (1–4). In the dog, left atrial enlargement is a common sequela in many cardiac diseases including left-sided congenital cardiac disease, myxomatous mitral valve disease (MMVD), and dilated cardiomyopathy (DCM) (7). Other risk factors for development of AF in humans include advanced age and congestive heart failure (CHF) (1, 2). In dogs with cardiac disease, few studies specifically investigated the risk factors of AF development. In addition to left atrial enlargement, investigators have identified bodyweight as a risk factor (7). Furthermore, two recent studies found that CHF, decreased left ventricular fractional shortening, and right atrial enlargement, increased risk of developing AF (8, 9). Furthermore, presence of AF decreased survival in medium to large-size dogs with MMVD and CHF (10) and increased the risk of cardiac-related death and decreased survival time in Dobermann pinschers with DCM and CHF (9). However, because MMVD and DCM predominantly affect dogs with different bodyweights, the prevalence of AF is low in dogs with MMVD (8) but much higher in dogs with DCM (11, 12). Different studies investigated the prevalence, etiology and clinical importance of AF in Irish wolfhounds (6, 13–17). However, no study has specifically investigated the risk factors of developing AF in a heterogeneous population of dogs of different breeds with DCM at different disease stage.

The aim of the present study was therefore to identify the risk factors for developing AF in dogs of different breeds with an echocardiographic diagnosis of DCM.

## 2. Materials and methods

### 2.1. Study design and animals

In this retrospective study, we searched the electronic databases of five cardiology referral centers for dogs with an echocardiographic diagnosis of DCM that were examined between January 2018 and December 2021. This diagnosis was based on use of breed-specific cut-off values for Doberman pinschers (18) and Boxer dogs (19) and use of normalized left ventricular (LV) internal dimensions using allometric scaling (20) and fractional shortening for dogs of the other breeds (21, 22). Specifically, Doberman pinschers were diagnosed with DCM when left ventricular end-diastolic volume (determined by a modified Simpson’s method of disks) normalized to body surface area (BSA) exceeded 95 mL/m^2^ and end-systolic volume normalized to BSA exceeded 55 mL/m^2^ (18). Boxer dogs were diagnosed with DCM when left ventricular diastolic diameter (LVDD), left ventricular systolic diameter (LVSD), and fractional shortening were > 4.8 cm, >3.3 cm and < 21%, respectively (19) in the absence of other obvious cardiac pathology. In the remaining dogs, DCM was diagnosed when body weight normalized LVDD (LVDDn), LVSD (LVSDn) measured on M-mode echocardiography were > 1.63 and > 0.92, respectively, and fractional shortening was <20% (20–22) in the absence of atrio-ventricular valve lesion (i.e., thickened or prolapsing leaflets).

We classified the DCM as preclinical (occult) for dogs without clinical signs of CHF, and clinical (overt) for dogs with clinical signs of CHF (e.g., tachypnea, respiratory distress, and abdominal distension). Furthermore, we recorded the type of CHF, namely left-sided CHF, right-sided CHF, and biventricular CHF, as previously described (23, 24).

The presence or absence of AF was diagnosed based on at least one of the following techniques: surface ECG recording with dedicated electrocardiographs for at least 3 min or good quality ECG recordings throughout the echocardiographic exam. In particular, we diagnosed AF during the echocardiographic exam when an irregularly irregular cardiac rhythm with narrow QRS complexes was found associated with an isoelectric trace without recognizable P waves, and absent A wave on M-mode imaging of the mitral valve and spectral Doppler interrogation of trans-mitral blood flow (8).

Dogs with prevalent sinus rhythm and dogs with cardiac arrhythmias other than AF were combined into the group of dogs without AF. Thus, only two groups of dogs were considered for analysis: dogs with AF and dogs without AF. We excluded dogs without cardiac rhythm evaluation and with an equivocal cardiac diagnosis, or dogs with concomitant congenital or acquired cardiac disease (e.g., those with thickened and/or prolapsing mitral valve leaflets). Dogs with hypothyroidism were included in the study if they were on treatment and the condition was considered stable at inclusion (25). Specifically, we excluded dogs with suspected myocarditis and tachycardia-induced cardiomyopathy but included dogs with suspected nutritionally related cardiomyopathies. We considered myocarditis as the most likely diagnosis when cardiac troponin I level (cTnI) was >1.0 ng/mL using a high sensitivity cTnI assay (26). Dogs with suspected tachycardia-induced cardiomyopathy were excluded when ventricular or supraventricular tachycardia other than AF was observed on routine or 24-h ECG.

### 2.2. Echocardiographic examination

Experienced operators performed complete echocardiographic examinations, including two-dimensional (2D) real time, M-mode, and echo-Doppler analysis with simultaneous ECG tracing at each center. The measurements of LVDD and LVSD were obtained from M-mode short-axis echocardiographic images at the level of *chordae tendinae.* Left ventricular diastolic and systolic measurements were then transformed using the described allometric scaling system (20) to obtain their normalized measurements (LVDDn and LVSDn, respectively). The fractional shortening was also calculated using the formula (LVDD-LVSD)/LVDD*100.

Measurements of the left atrial (LA) diameter and aortic diameter (Ao) were obtained from right parasternal 2D short axis images of the heart base at the first frame after aortic valve closure (i.e., early diastole) and used to calculate the LA-to-Ao ratio (LA:Ao) (27, 28). We identified right atrial enlargement by subjective comparison to LA size on right parasternal 2D long axis images (9). The trans-mitral blood flow was examined using pulsed-wave Doppler from the left apical four-chamber view and the peak velocity of early trans-mitral diastolic blood flow (Emax) was obtained. Presence of tricuspid regurgitation was searched using different echocardiographic views and, when present, the peak velocity was measured using continuous-wave Doppler.

All echocardiographic measurements were obtained by averaging measurements of at least three and five consecutive beats for dogs without and with AF, respectively.

### 2.3. Statistical analysis

Data were analyzed using different commercial software’s (IBM SPSS Statistics for Windows, Version 24.0. Armonk, NY: IBM Corp.; MedCalc Statistical Software version 16.4.3, MedCalc Software, Ostend, Belgium). Demographic and clinical characteristics included breed, sex, age, bodyweight, presence and type of CHF, ongoing treatment at admission, and presence of concurrent diseases. For breed and type of CHF, the following categories were considered, respectively: Great Dane, Dobermann pinscher, Labrador retriever, and other breeds; and left-sided CHF, right-sided CHF, and biventricular CHF. The following continuous echocardiographic variables were considered: LA diameter, Ao, LA:Ao, LVDDn, LVSDn, fractional shortening, and Emax. Furthermore, presence or absence of right atrial enlargement and tricuspid regurgitation were used as binary variables.

Continuous data were assessed using the Shapiro–Wilk’s test for normality and reported as mean and standard deviation, while categorical variables were presented as number and percentage within each category. The comparison of clinical and echocardiographic variables between dogs developing AF and those not developing AF was carried out using the Student’s t-test and the two proportions zeta test for continuous and categorical variables, respectively.

Univariate and stepwise multivariable logistic regression analysis was used to estimate the odds ratio (OR) with 95% confidence interval (CI) of developing AF. Variables significantly associated with AF with *p* < 0.05 in the univariate analysis were entered in the final multivariable model using the stepwise backward elimination method. Receiver operating characteristic (ROC) curve analysis was carried out for continuous variables showing significant correlation with development of AF in the univariate logistic regression analysis (i.e., bodyweight, LA diameter, Ao, LA:Ao, and Emax) to evaluate their accuracy to predict development of the arrhythmia. In particular, the area under the curve (AUC), with corresponding 95% CI, as well as the sensitivity, specificity, and positive and negative likelihood ratio, were calculated at various cut-off points. For all analyses, the significance was set for *p* < 0.05.

## 3. Results

### 3.1. Animals

We identified 126 dogs with echocardiographic DCM. We excluded 31 dogs because of non-compliance with the echocardiographic criteria and six because of high cTnI concentrations. Thus, we included 89 dogs in the analyses, consisting of 71 males (80%) and 18 females (20%). Nineteen different breeds comprised the sample population, including 39 Dobermann pinschers (44%), 11 Labrador retrievers (12%), 8 Great Danes (9%), 6 mixed breed dogs (7%), 4 Boxers (5%), and three Cane Corso, Cocker spaniels, and German shepherds (3%). Other breeds were represented by two or fewer dogs. Dogs had a mean age of 93 ± 32 months (range: 24 to 154 months); mean bodyweight of 40 ± 13 kg, with only 3 dogs (3%) weighing less than 20 kg. Seventy-three dogs (82%) had CHF, including 39 dogs with left-sided CHF (53%), 11 dogs with right-sided CHF (15%), and 23 dogs with biventricular CHF (32%). Of these, 39 dogs (44%) were receiving one or more drugs for the treatment of CHF and 25 dogs (28%) had concurrent non-cardiac diseases.

Atrial fibrillation was diagnosed as the prevalent cardiac rhythm in 39 dogs (44%), whereas 29 dogs (33%) exhibited a sinus rhythm. The remaining 21 dogs (24%) showed other types of cardiac arrhythmias, including 18 dogs (20%) with ventricular arrhythmias, two dogs (2%) with supraventricular arrhythmias other than AF, and one dog (1%) with second degree atrio-ventricular block. Higher proportions of Great Danes (*p* = 0.013), dogs with CHF (*p* = 0.012), and dogs with right atrial enlargement (*p* < 0.001) and tricuspid regurgitation (*p* = 0.013) had AF than dogs without AF, whereas left-sided CHF was more prevalent in dogs without AF (*p* = 0.013) (Table 1). Dogs with AF were heavier (*p* < 0.001), had higher heart rates (*p* < 0.001), larger LA and Ao diameters (*p* < 0.001 and *p* = 0.001, respectively), larger LA:Ao (*p* = 0.015), and higher Emax (*p* = 0.014) (Table 1) compared to dogs without AF; mean age did not differ (*p* = 0.132).

**Table 1:** Descriptive data obtained from 89 dogs with dilated cardiomyopathy divided in two groups based on presence or absence of atrial fibrillation.

Variable	Category	Total (*N* = 89)	AF group (*N* = 39)	No AF group (*N* = 50)	*p* value
		Number (%), mean ± SD	Number (%), mean ± SD	Number (%), mean ± SD	
Breed	Great Dane	8 (9.0)	7 (18.0)	1 (2.9)	0.013
	Dobermann pinscher	39 (43.8)	13 (33.3)	26 (52.9)	0.122
	Labrador retriever	11 (12.4)	3 (7.7)	8 (16.0)	0.274
	Other	31 (34.8)	16 (41.0)	15 (30.0)	0.390
Sex	Male/Female	71/18 (79.8/20.2)	35/4 (89.7/10.3)	36/14 (72.0/28.0)	0.072
Age (month)	mean ± SD	93 ± 32	99 ± 30	88 ± 33	0.132
BW (kg)	mean ± SD	40 ± 13	46 ± 13	35 ± 10	< 0.001
CHF	All types	73 (82.0)	37 (94.9)	36 (72.0)	0.012
	L-CHF	39 (53.4)	14 (37.8)	25 (69.5)	0.013
	R-CHF	11 (15.1)	8 (21.6)	3 (8.3)	0.112
	B-CHF	23 (31.5)	15 (40.6)	8 (22.2)	0.152
Concurrent diseases	Yes/No	25/64 (28.1/71.9)	10/29 (25.6/74.4)	15/35 (30.0/70.0)	0.829
Treatment at admission	Yes/No	39/50 (43.8/56.2)	20/19 (51.3/48.7)	19/31 (38.0/62.0)	0.299
Heart rate (bpm)	mean ± SD	158 ± 45	193 ± 31	131.06 ± 34.32	< 0.001
LA (cm)	mean ± SD	4.91 ± 1.16	5.6 ± 0.93	4.37 ± 1.04	< 0.001
Ao (cm)	mean ± SD	2.48 ± 0.46	2.67 ± 0.45	2.34 ± 0.42	0.001
LA:Ao	mean ± SD	2.00 ± 0.45	2.13 ± 0.4	1.90 ± 0.46	0.015
LVDDn	mean ± SD	2.07 ± 0.25	2.05 ± 0.24	2.08 ± 0.25	0.511
LVSDn	mean ± SD	1.29 ± 0.17	1.27 ± 0.17	1.30 ± 0.18	0.465
FS (%)	mean ± SD	13.56 ± 4.84	12.67 ± 5.26	14.25 ± 4.4	0.126
E mitral (m/s)	mean ± SD	1.08 ± 0.39	1.19 ± 0.41	0.98 ± 0.36	0.014
RAE	Yes/No	37/52 (41.6/58.4)	25/14 (64.1/35.9)	12/38 (24.0/76.0)	< 0.001
TR	Yes/No	52/37 (58.4/41.6)	29/10 (74.4/25.6)	23/27 (46.0/54.0)	0.013

Data are expressed as mean ± standard deviation (SD) and number (n.) and (percentage). N, number of dogs; BW, body weight; CHF, congestive heart failure; LA, left atrial diameter; Ao, aortic diameter; LA:Ao, left atrial diameter to aortic diameter; LVDDn, left ventricular diastolic diameter normalized to body weight; LVSDn, left ventricular systolic diameter normalized to body weight; FS, Fractional shortening; E mitral, mitral valve maximal E wave velocity; R, right; RAE, right atrial enlargement; TR, tricuspid regurgitation; L-CHF, left-sided CHF; R-CHF, right-sided CHF; B-CHF, biventricular CHF.

### 3.2. Logistic regression and ROC curve analysis

Univariable logistic regression showed that presence of AF was positively correlated with being a Great Dane compared to Dobermann pinscher and Labrador retriever (*p* = 0.019 and *p* = 0.021, respectively), male sex (*p* = 0.046), bodyweight (*p* < 0.001), CHF (*p* = 0.013), LA diameter (*p* < 0.001), Ao (*p* = 0.002), LA:Ao (p = 0.019), Emax (*p* = 0.019), presence of right atrial enlargement and tricuspid regurgitation (*p* < 0.001 and *p* = 0.008, respectively, Table 2). In the final multivariable model only increased LA diameter (OR = 3.58, 95% CI 1.87–6.87; *p* < 0.001) and presence of right atrial enlargement (OR = 4.02, 95% CI 1.35–11.97; *p* = 0.013) were identified as independent risk factors for developing AF (Table 3).

**Table 2:** Results of the univariate logistic regression analysis showing the association between the risks for developing atrial fibrillation in 89 dogs with dilated cardiomyopathy.

Variable	Chi-square	Odds ratio	95%CI	*p*
Breed	7.79	NA	NA	0.050
Dobermann pinscher vs. Great Dane	5.53	0.07	0.01–0.64	0.019
Labrador retriever vs. Great Dane	5.35	0.05	0.01–0.64	0.021
Other breeds vs. Great Dane	2.78	0.15	0.02–1.39	0.095
Sex (male)	3.97	3.40	1.02–11.35	0.046
Age (months)	2.26	1.01	1.00–1.02	0.133
BW (kg)	12.96	1.09	1.04–1.14	<0.001
Treatment at admission	1.75	1.79	0.75–4.28	0.186
Concurrent diseases	0.20	0.80	0.31–2.06	0.650
CHF	6.22	7.19	1.52–33.93	0.013
LA (cm)	18.82	3.94	2.12–7.34	<0.001
Ao (cm)	9.55	5.92	1.92–18.26	0.002
LA:Ao	5.54	3.43	1.23–9.56	0.019
LVDDn	0.27	0.56	0.10–3.11	0.507
LVSDn	0.54	0.40	0.03–4.68	0.461
FS (%)	2.32	0.93	0.85–1.02	0.127
E mitral (m/s)	5.50	4.34	1.27–14.82	0.019
RAE	13.58	5.66	2.25–14.21	<0.001
TR	6.98	3.40	1.37–8.45	0.008

BW, body weight; CHF, congestive heart failure; LA, left atrial diameter; Ao, aortic diameter; LA:Ao, left atrial diameter to aortic diameter ratio; LVDDn, left ventricular diastolic diameter normalized to body weight; LVSDn, left ventricular systolic diameter normalized to body weight; FS, Fractional shortening; E mitral, mitral valve maximal E wave velocity; RAE, right atrial enlargement; TR, tricuspid regurgitation; NA, not applicable.

**Table 3:** Results of the multivariable logistic regression analysis showing the association between the risks for developing atrial fibrillation in 89 dogs with dilated cardiomyopathy.

Variable	Chi-square	Odds ratio	95%CI	*p*
LA (cm)	14.8	3.58	1.87–6.87	<0.001
RAE	6.23	4.02	1.35–11.97	0.013

LA, left atrial diameter; RAE, right atrial enlargement.

**Figure 1:**
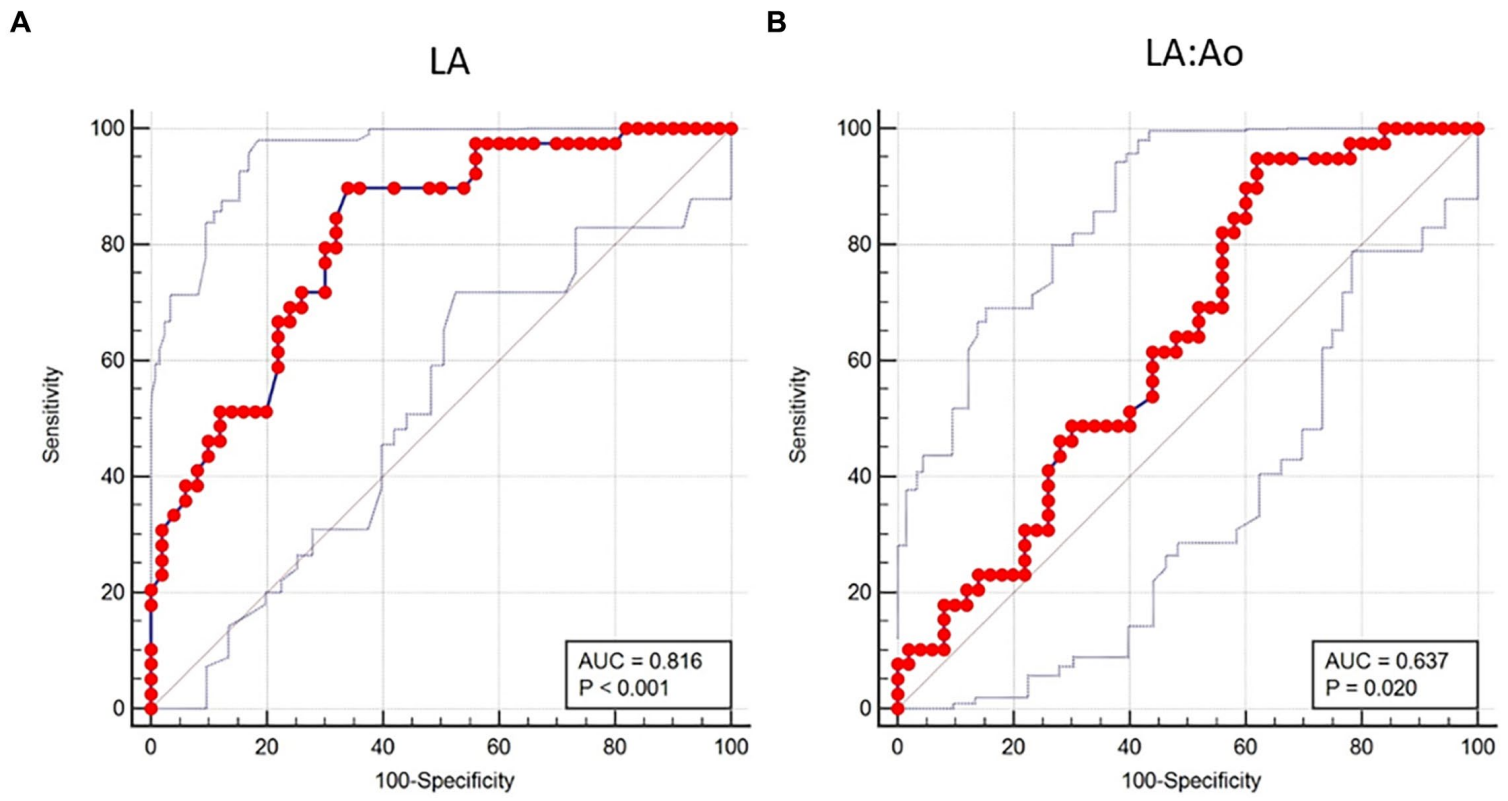
Receiver operating characteristic curve of two echocardiographic methods of left atrial measurement for the prediction of development of atrial fibrillation in a population of 89 dogs with dilated cardiomyopathy. **(A)** The area under the curve of the left atrial diameter (LA) is 0.816 ± 0.044 (95% confidence interval [CI] = 0.719–0.890) at the cut-off of >4.66 cm. **(B)** The area under the curve of the left atrial-to-aortic root diameter ratio (LA:Ao) is 0.637 ± 0.059 (95% CI = 0.528–0.736) at the cut-off of >1.73.

The LA diameter (AUC = 0.81, 95% CI 0.72–0.89) had the highest accuracy to predict development of AF at the cut-off >4.66 cm with sensitivity and specificity of 89.7 and 66.0%, respectively (Table 4; Figure 1). Body weight and LA:Ao showed moderate accuracy in predicting the presence of AF (AUC = 0.74, 95% CI 0.64–0.83 and AUC = 0.64, 95% CI 0.53–0.70) at the cut-off of >36 kg and > 1.73, respectively.

**Table 4:** Diagnostic accuracy of five clinical and echocardiographic variables to predict development of atrial fibrillation in 89 dogs with dilated cardiomyopathy.

Variable	AUC ± SE	95% CI	Cutoff	Sensitivity	Specificity	+LR	−LR
BW (kg)	0.740 ± 0.052	0.636–0.827	>36	0.79	0.58	1.89	0.35
LA (cm)	0.816 ± 0.044	0.719–0.890	>4.66	0.90	0.66	2.64	0.16
Ao (cm)	0.686 ± 0.056	0.579–0.780	>2.3	0.85	0.50	1.69	0.31
LA:Ao	0.637 ± 0.059	0.528–0.736	>1.73	0.95	0.38	1.53	0.13
E mitral (m/s)	0.647 ± 0.059	0.536–0.748	>0.79	0.89	0.42	1.53	0.26

BW, body weight; LA, left atrial diameter; Ao, aortic diameter; LA:Ao, left atrial diameter to aortic diameter ratio; E mitral, mitral valve maximal E wave velocity; AUC, area under the curve; SE, standard error; CI, confidence interval; +LR, positive likelihood ratio; −LR, negative likelihood ratio.

## 4. Discussion

Our study confirms that AF occurs commonly in large-breed dogs with DCM, with the biggest predictors of the presence of AF on presentation being the size of the left atrium and evidence of right atrial enlargement. While bodyweight, breed, sex, presence of CHF, and LA:Ao independently predicted presence of AF, these variables dropped out of the model when LA diameter (absolute) and right atrial enlargement (subjective) were included.

Our results contrast those of previous investigators, who reported higher prevalence AF in more selected populations of dogs with DCM, including 48% of Dobermann pinschers with DCM and CHF (9), and ranging from 77 to 88% of Irish wolfhounds with DCM (13, 15). However, Irish wolfhound are particularly prone to developing AF even in the absence of overt DCM (6, 17), and we included no Irish wolfhounds in the present study.

Our results showed that dogs with DCM and AF differed clinically and echocardiographically from those without AF. Great Danes with AF outnumbered those without AF, but we did not find a similar predominance for the other two most represented canine breeds in this study (Dobermann pinschers and Labrador retrievers). Furthermore, AF was more prevalent in heavier dogs, but we found no difference based on sex or age, similar to previous observations of higher prevalence of AF in large and giant breed dogs with DCM (e.g., Great Dane and Newfoundland) than in smaller dogs (e.g., Cocker spaniels) (12). This differs from findings in humans, where both age and gender (male) increase the risk of developing AF (2). Other investigators, examining the dog population admitted to 26 colleges of veterinary medicine in the USA for any reason, reported large differences in AF prevalence based on breed and associated bodyweight, ranging from 0.04% in Miniature poodles to 5.84% in Irish wolfhound (29). While our results confirmed the higher prevalence of DCM in male dogs compared to females (11, 12), we did not identify an increased prevalence of AF in male dogs. This differs from studies in humans and dogs with MMVD that identified a higher percentage of AF in males (2, 8).

Dogs with AF had higher heart rate than those without AF, consistent with previous observations of dogs with AF and MMVD (8), but we found no differences regarding presence of concurrent diseases and treatment at admission. In humans, different non-cardiac diseases are recognized risk factors for incident AF, including diabetes mellitus, chronic kidney disease, and chronic obstructive pulmonary disease (2), but other comorbidities do not appear to increase risk of AF in the dog (8, 29).

Dogs with AF had significantly increased Emax, LA diameter, Ao, and LA:Ao. These findings suggest that either absolute LA size (expressed by LA diameter) and relative increase in LA size (expressed by LA:Ao ratio) could be determinant for development of AF in dogs with DCM. Increased left atrial pressure, indirectly estimated through increased Emax (30) could also predispose to development of AF. Furthermore, AF was found in a significantly higher percentage of dogs with CHF, but not in those with left-sided CHF, as well as in animals with right atrial enlargement and tricuspid regurgitation. All these factors suggest a more advanced stage of DCM.

Univariable logistic regression showed that Great Danes (compared to Dobermann pinschers and Labrador retrievers), male dogs, larger dogs, dogs with larger LA diameter, Ao, LA:Ao, and higher Emax, and dogs with right atrial enlargement and CHF had increased risk of developing AF. However, after the multivariable analysis, only LA diameter and right atrial enlargement remained as predictors of probability of presenting with AF. These findings suggest that increased dimensions of both atria are the only independent risk factors for presenting with AF in dogs with DCM. Specifically, per each cm increase in LA diameter and if right atrial enlargement is present, there is an approximately three-and-a-half and four-fold increased risk of developing AF, respectively. Because of their higher bodyweight compared to dogs with MMVD, only absolute LA dimension but not the most commonly employed echocardiographic index of LA enlargement, namely LA:Ao, is a useful predictor of developing AF in dogs with DCM. Furthermore, bodyweight was not retained as an independent risk factor for AF development in dogs of the present study. These findings suggest that bodyweight and LA diameter should still be considered risk factors for developing AF in the general population of dogs with cardiac disease associated with left atrial enlargement. However, when considering the more restricted population of dogs with DCM, the majority of them weighing more than 20 kg, the bodyweight become a less important factor, as do echocardiographic indices of relative left atrial enlargement (e.g., LA:Ao). Presence of right atrial enlargement, but not of CHF, also independently predicted presence of AF in dogs of the present study, as recently reported in Dobermann pinschers with DCM and CHF (9). While numerous studies evaluated the importance of LA structural changes in subjects with AF, the role of right atrial enlargement has been poorly investigated in both humans (31) and dogs.

Results of ROC curve analysis regarding LA dimension showed that a cut-off value of 4.66 cm for LA diameter had the highest accuracy to predict presence of AF in dogs of the present study. Lower accuracy were found for bodyweight and LA:Ao. These findings further confirm the importance of absolute LA dimensions for developing AF in dogs with DCM. Although LA:Ao at the cut-off of 1.73 had higher sensitivity compared to LA diameter to predict presence of AF (95% versus 90%), its specificity was much lower (38% versus 66%). In humans with embolic stroke of undetermined etiology (32), and in dogs with MMVD (8), a LA diameter threshold of 40 mm and 3.45 cm, respectively, had the best performance to predict development of AF. Underlying heart disease causing chronic stretch is a known cause of atrial structural remodeling (33), but species-related and disease-related factors can also play an important role to determine specific threshold predictive of AF development. In particular, a higher LA diameter threshold must be achieved to induce development of AF in dogs with DCM compared to those with MMVD, because many of the large breed dogs have LA diameter that exceeds 3.45 cm without exhibiting AF. However, it must be highlighted that atrial dilation can be both a cause and consequence of AF (34), as observed in Irish wolfhounds where AF has been shown to be a precursor to cardiac chamber dilation and DCM (14, 15).

Atrial structural remodeling, including both macroscopic and microscopic changes, is the key factor underlying all AF-related mechanisms (35, 36). Atrial dilatation and fibrosis are the hallmarks of macroscopic and microscopic changes during atrial remodeling in people with AF (35, 37). Furthermore, fibro-fatty infiltration of the sub-endocardium could also contribute to atrial myocardium fibrosis in patients with AF (38). In the dog, a study evaluated and compared the histopathologic pattern of the left atrium in animals with DCM and MMVD, 94 and 7% of them presenting AF, respectively (39). Left atrial myocardial pathology in dogs with DCM differed from that in dogs with MMVD, including more interstitial but less perivascular fibrosis, less distinct vessel narrowing, and more strongly defined degenerative changes (38). In Irish wolfhounds, right atrial appendages from dogs with AF with or without DCM had significantly more myocardial fibrosis and adipocyte infiltration compared with normal hearts (16). Furthermore, fibro-fatty replacement of the atrial myocardium was described in a large breed dog with MMVD and AF (40). Thus, atrial fibrosis and fibro-fatty replacement seem to play an important role in atrial remodeling of dogs with AF, but this association deserves further studies.

Heart failure is another demonstrated risk factors for incident AF in humans (2) and these two cardiovascular conditions often coexist although it is not yet clear whether AF begets CHF or vice versa (41) posing the classic chicken or the egg causality dilemma. In particular, AF precedes and follows CHF in patients with both preserved and reduced ejection fraction (41), in other words subjects with preserved or reduced systolic function. Presence of CHF was not an independent risk factor for developing AF in dogs of the present study, differently from those with MMVD (8). These findings could suggest that CHF precedes AF in dogs with preserved systolic function, as is the majority of dogs with MMVD, but not in those with reduced systolic function, namely dogs with DCM. However, reduced fractional shortening was also a risk factor of developing AF in dogs with MMVD (8). Results of fractional shortening measurements should be cautiously interpreted in dogs with MMVD, but reduced fractional shortening suggests reduced systolic function in these animals (42). Thus, the precise cause-effect relationship between AF and CHF remain uncertain in both dogs and humans (8, 43), also because the temporal relationship of each condition cannot be exactly determined, particularly in dogs. Together, AF and CHF portend a poor prognosis in both species (9, 10, 41).

This study has some inherent limitation because of the retrospective design. Canine DCM can result from various etiologies, including genetic mutations, toxins, infections, and nutritional imbalances (44), and it is characterized by morphologic or electrical cardiac derangement, or both (22). We only included dogs with an echocardiographic diagnosis of DCM and excluded those with exclusive cardiac electrical derangement. Thus, our results should be only interpreted in the context of the former group of dogs. Furthermore, the echocardiographic diagnosis of DCM as well as differentiation of primary DCM from tachycardia-induced DCM in dogs with AF is challenging, as recently highlighted in an elegant review article (21). We used a simplified approach to diagnose DCM in dogs of the present study because of the variability of echocardiographic measurements obtained in different veterinary centers in dogs of various breeds. However, we followed some of the most recently available recommendations to diagnose this multifaceted canine myocardial disease (18, 21, 22). We based our cardiac rhythm evaluation either on ECG recording using dedicated electrocardiographs or on good quality ECG inspection during the echocardiographic exams. Although this latter can represent a less reliable method of cardiac rhythm analysis, nevertheless it allows recognition of the preeminent AF characteristics, including isoelectric trace without evident P wave associated with irregularly irregular cardiac rhythm with narrow QRS complexes, and absent A wave on M-mode imaging and spectral Doppler interrogation of left ventricular diastolic inflow (8). Echocardiographic evaluation of atrial dimension was based on linear measurement and subjective evaluation for the left and right atrium, respectively. More accurate and specific methods of measurement have been proposed for both atria (45–47). However, measurement of the LA diameter in a short axis plane with calculation of LA:Ao and subjective evaluation of right atrial dimension represent the most commonly employed methods for diagnosing atrial enlargement in the veterinary clinical practice (21). In addition to LA enlargement, LA dysfunction is associated with AF development and can be identified using other echocardiographic techniques and indices (e.g., LA active filling, total fractional area change, and LA strain using speckle tracking echocardiography) (48, 49). Furthermore, LA strain can provide useful predictive information for AF development in both humans and dogs (49–51), however, we used no advanced echocardiographic techniques, including speckle tracking echocardiography evaluation of LA strain in the present study. Speckle tracking echocardiography has only been used for the assessment of LA function in healthy dogs and dogs with MMVD (52–58), but not so far in dogs with DCM. Finally, the small sample size of some demographic (i.e., breed and sex) and clinical variables (i.e., type of CHF) may have influenced the results of their effect on the development of AF.

## 5. Conclusion

In conclusion, AF and CHF are commonly associated with DCM in dogs. Increased right atrial dimension and LA enlargement, echocardiographically assessed by measuring the LA diameter but not LA:Ao, are independent risk factors for the presence of AF in these dogs. In contrast, previously demonstrated risk factors for the development of AF in humans (i.e., age and CHF) and small-breed dogs with MMVD (i.e., bodyweight and CHF) do not play a similar role in large-breed dogs with DCM.

## Data availability statement

The raw data supporting the conclusions of this article will be made available by the authors, without undue reservation.

## Ethics statement

Ethical review and approval was not required for the animal study because In accordance with the European Parliament and Council normative 2010/63/UE (22nd September 2010) on the protection of animals used for scientific purposes and with the National Guidelines of Italian Ministry of Health for the care and use of animals (D.L. 4 March 2014 n. 26 and D.L. 27 January 1992 n.116) the non-experimental clinical veterinary practice is excluded from the scope of legislation and therefore ethical approval was not required for this study. Every clinical assessment and procedure were necessary and performed in the interest of dogs’ health. Written informed consent was obtained from the owners for the participation of their animals in this study.

## Author contributions

CG and HP: conceptualization. BC: formal analysis. CV, CG, MB, GR, CM, GM, MW, TB, OD, VP, FP, PF, DC, and HP: investigation and data collection. CG and CV: writing—original draft preparation. CV, CG, MB, GR, GM, OD, FP, and HP: writing—review and editing. All authors contributed to the article and approved the submitted version.

## Funding

This work was supported by a grant of the University of Padua, Italy, to CG (SID Year: 2021-Project code C25F21000830001).

## Conflict of interest

The authors declare that the research was conducted in the absence of any commercial or financial relationships that could be construed as a potential conflict of interest.

## Publisher’s note

All claims expressed in this article are solely those of the authors and do not necessarily represent those of their affiliated organizations, or those of the publisher, the editors and the reviewers. Any product that may be evaluated in this article, or claim that may be made by its manufacturer, is not guaranteed or endorsed by the publisher.
